# Late‐Onset Vitiligo: Epidemiology, Clinical Characteristics, and Management Strategies

**DOI:** 10.1111/jocd.16705

**Published:** 2024-11-28

**Authors:** Zahbi Hasan, Yashdeep Singh Pathania

**Affiliations:** ^1^ Department of Dermatology, Venereology and Leprology All India Institute of Medical Sciences Rajkot Gujarat India

**Keywords:** adult‐onset vitiligo, late‐onset vitiligo, vitiligo in adults, vitiligo in elderly

## Abstract

**Background:**

Late‐onset vitiligo (LOV), generally defined as vitiligo that starts at age 30 or older, presents unique diagnostic and management challenges, reflecting an intricate interplay of genetic, environmental, and societal factors.

**Objectives:**

This review aims to elucidate the distinct aspects of LOV such as epidemiology, clinical characteristics, and treatment outcomes thereby enhancing diagnostic precision and planning management strategies.

**Materials and Methods:**

A comprehensive literature search was conducted across multiple databases including PubMed and EMBASE, adhering to PRISMA guidelines. Studies focused on adults (age 30 or older at the time of diagnosis) with LOV were included. Data on demographics, clinical features, and comorbidities were extracted.

**Results:**

The literature search yielded five eligible articles with a total sample size of 1099 patients. LOV prevalence ranged from 6.5% to 14.7%, with a mean age of onset in the mid to late 50s. Vitiligo vulgaris was the most common form, with increased leukotrichia and the Koebner phenomenon. Associated autoimmune/endocrine disorders, including diabetes mellitus and thyroid diseases, were prevalent, suggesting systemic links. Treatment outcomes varied, with combination therapy and phototherapy showing promise.

**Conclusion:**

Late‐onset vitiligo differs significantly from early‐onset vitiligo in its clinical traits, epidemiology, and treatment response, necessitating personalized care and targeted management strategies.

To the Editor

While there is no universally accepted definition for late‐onset vitiligo (LOV), it is often categorized as occurring at age 30 or older, with some studies defining it as age 50 and older [1]. This systematic review aims to highlight a critical need for a more systematic understanding of LOV to evaluate and improve diagnostic accuracy and patient management.

Following Prisma guidelines, databases, such as PubMed, Google Scholar, EMBASE, Cochrane Library, and Web of Science, were searched to date using search terms including “late‐onset vitiligo,” “adult‐onset vitiligo,” “vitiligo in old population,” and “vitiligo in elderly” (Figure 1).

**FIGURE 1:**
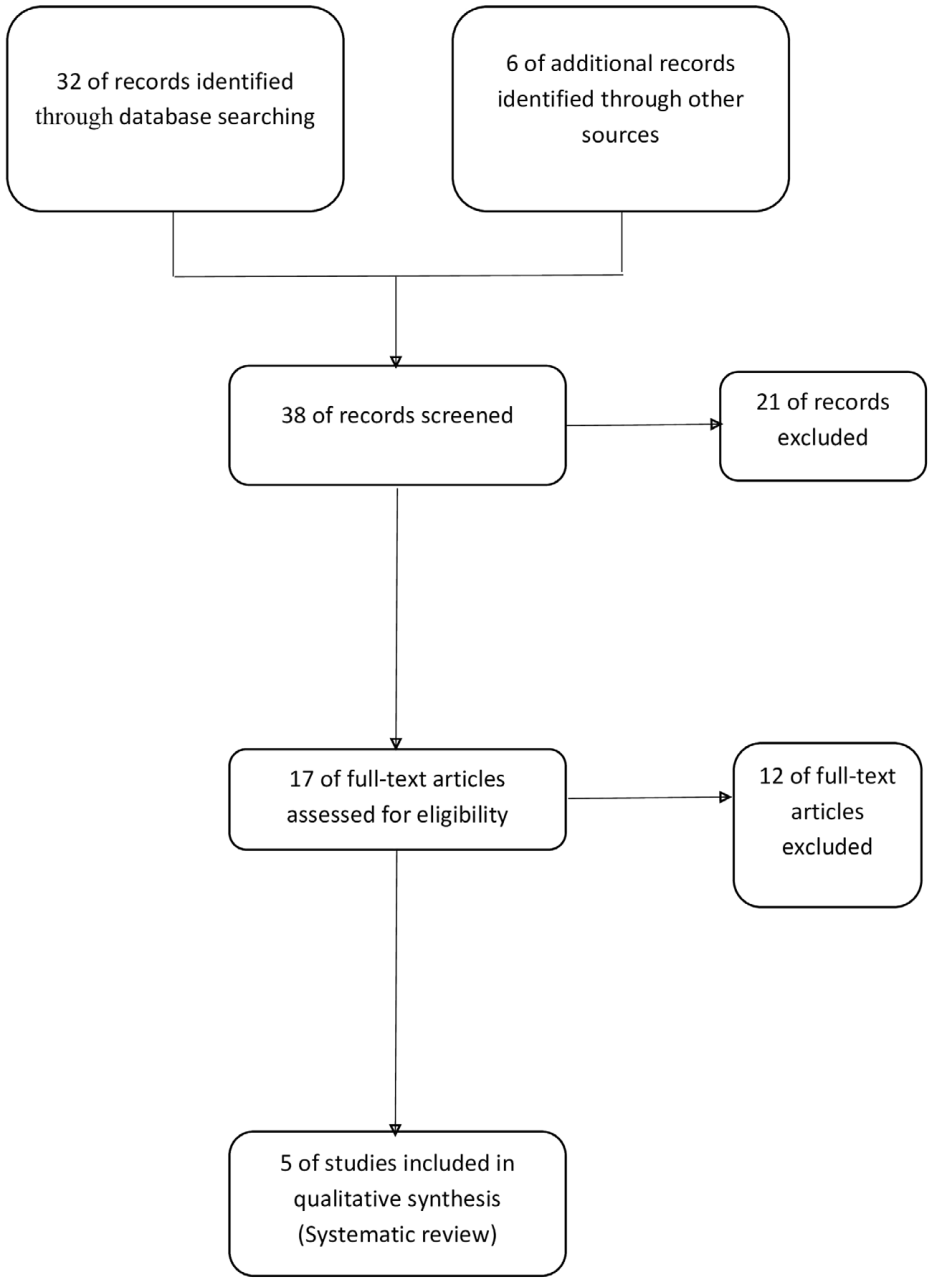
Study flow diagram.

Late‐onset vitiligo (LOV) prevalence varied by region from Iran to South East Asia, ranging from 6.5% to 14.7%, respectively. The average age of onset was found to range from mid to late 50s across all the studies, with slight regional variations with the mean age of onset ranging from 52.5 to 59.4 ± 7.4 years. Gender distribution is nearly equal with most studies reporting almost balanced gender ratios, while Kong et al. observed a slight female predominance (56.4%) [2].

Vitiligo vulgaris was the most common form ranging from 59.3% to 83.5%. Focal vitiligo was predominantly observed in the Kong et al. study, accounting for 45.3% of their cases. The head and neck (Dogra et al.) [3], upper limbs (Al‐Mutairi et al.) [4], and face (Kong et al.) [2] were reported as frequent initial sites of onset. Leukotrichia (72%–77.8%) and Koebner's phenomenon (79.6%) were notably higher in late‐onset groups [4]. Disease stability in the form of no spread for 2 years in majority of the patients was a notable feature of LOV [3]. The prevalence of associated autoimmune/endocrine disorders ranging from 18.5% to 27.41%, with diabetes mellitus (11.1%–27.41%), thyroid diseases (1.9%–9.64%), alopecia areata (3.7%), rheumatoid arthritis, and other conditions like pernicious anemia and Addison's disease being observed. Nonautoimmune disorders like hypertension, seizure disorders, and polycystic ovary disease were also noted in some studies. This suggests a strong link between LOV and systemic diseases, possibly due to shared autoimmune pathologies. Similar diseases in the family varied from 23.2% to 27.8% [5]. Kong et al. provided the most focused data on treatment response, suggesting good outcomes with combination therapy utilizing topical medications and phototherapy, notably in the focal vitiligo subtype. The study also noted the effectiveness and tolerance of phototherapy in the elderly late‐onset vitiligo population.

This review provides valuable data for future research on the differences between late‐onset and early‐onset vitiligo. Longitudinal studies could deepen understanding of LOV's progression, stability, and treatment responses, informing optimal management strategies. Clinically, these findings underscore the need for personalized care that accounts for LOV's unique characteristics and comorbidity profiles. Additionally, educational programs for clinicians on these distinctions could enhance diagnostic precision, treatment outcomes, and patient quality of life.

The late‐onset vitiligo differed significantly from early‐onset vitiligo in its clinical traits, epidemiology, and treatment response. This review highlights the importance of recognizing these distinctions for a holistic approach to effective management and better outcomes (Table 1).

**TABLE 1:** Characteristics of late‐onset vitiligo.

Study	Demography	Prevalence	Clinical types	Predominant sites
Kong et al. [2]	South East Asia	14.7%	NSV, focal vitiligo	Face, extremities
2 Dogra et al. [3]	South East Asia	6.8%	NSV, vitiligo vulgaris	Head and neck
3 Esfandiarpour et al. [4]	Iran	6.5%	NSV, vitiligo vulgaris	Limbs
4 Al‐Mutairi et al. [5]	South East Asia	—	NSV, vitiligo vulgaris	Face, limbs

Abbreviation: NSV, nonsegmental vitiligo.

## Author Contributions

Zahbi Hasan: prepared the manuscript. Yashdeep Singh Pathania: finalized the manuscript and overall supervision.

## Consent

The authors have nothing to report.

## Conflicts of Interest

The authors declare no conflicts of interest.

## Data Availability

The authors have nothing to report.
